# Feline-Ality™ in Real Life: A Program Evaluation

**DOI:** 10.3390/ani13172752

**Published:** 2023-08-29

**Authors:** Saethra Darling

**Affiliations:** 1 Department of Psychology, University of Washington, Seattle, WA 98195, USA; darlings@oregonstate.edu; 2 Department of Animal and Rangeland Sciences, Oregon State University, Corvallis, OR 97331, USA

**Keywords:** domestic cats, adoption success, animal shelters, cat personality, owner expectations, cat behavior

## Abstract

**Simple Summary:**

Domestic cats are euthanized at high rates in shelters. Previous research suggests that appropriate matching between cats and their adopters increases adoption success and decreases euthanasia. The ASPCA’s Meet Your Match^®^ Feline-ality™ program is designed to improve adoption success by matching cats with owners based on measures of cat personality and owner lifestyle and expectations. However, despite having been implemented in many shelters, there has not been a systematic review of the implementation process and efficacy of the Feline-ality™ program. This study will describe process and outcome evaluations of Feline-ality™, following its 2017 implementation at the Seattle Animal Shelter in Seattle, WA (USA).

**Abstract:**

Domestic cats are euthanized at high rates in shelters, and appropriate matching between cats and adopters is believed to improve adoption success and reduce euthanasia rates. The ASPCA’s Meet Your Match^®^ Feline-ality™ program, designed to match cats with owners based on personality and lifestyle, has been implemented in various shelters. This study is the first systematic evaluation of its implementation process and efficacy. Using a controlled interrupted time series design (CITS), the outcome evaluation examined and compared records for five years (2015–2019) from the Seattle Animal Shelter (SAS) and the Multnomah County Animal Shelter located in Portland, Oregon (USA). The outcome evaluation demonstrated no changes in any outcomes (e.g., cat adoptions, euthanasias, returns, transfers, or length of stay) that could be reliably attributed to Feline-ality™. The process evaluation at SAS identified and quantified eight possible errors in implementation that could affect the success of Feline-ality™; the results indicated a 1.6% overall success rate. Whereas the lack of substantive effect may indicate that MYM^®^ Feline-ality™ does not perform as purported, it is equally as likely (if not more so) that this absence of evidence of any results reasonably attributable to Feline-ality™ has occurred because the error rate in implementation of the program was very high. The fact that a poorly implemented program necessarily impacts the results of an outcome evaluation underscores the need for process evaluation concurrent with implementation.

## 1. Introduction

Program evaluation is an important component of applied animal behavior science that is seldom found in the published literature [1]. Despite the popularity of evidence-based practice in virtually all arenas of organizational life, program evaluation is less common than it should be. For example, a 2017 government-wide report from the Government Accountability Office describes the implementation of program evaluation in only approximately 40% of governmental programs in the United States [2]. Information about the rate of program evaluation in private or not-for-profit entities is difficult to estimate, and even program evaluations in the public domain are not widely disseminated in scientific literature. In applied animal behavior science—where the main goals are to improve the care, management, and welfare of animals—there is a dearth of available data on the evaluation of programs instituted by the largest animal welfare organizations.

Many large and influential animal welfare organizations, including the American Society for the Prevention of Cruelty to Animals (ASPCA), have their own applied animal behavior research teams that not only conduct and publish applied research on domestic animals, but also use the results of their work to inform their own policies and practices. Specifically, as applied to the area of animal sheltering, one such program is the ASPCA’s Meet Your Match^®^ (MYM^®^). Originally designed to pair potential adopters with available pet dogs, the program was extended to include pet cats in 2007 and modified into its current form in 2015 [2,3]. The research conducted in designing the feline side of the program was not published in a peer-reviewed journal; a brief summary of that research can be found in the ASPCA’s MYM^®^ Feline-ality™ training manual [3]. A modification of the original Feline-ality™ program was undertaken in 2015 in response to logistical concerns from participating shelters (e.g., 72 h hold before assessment could be performed) and a comparison of the modified program and the original program was published [4]. No further work evaluating the implementation of (process evaluation) or effects of (outcome evaluation) the MYM^®^ Feline-ality™ program has been published subsequent to its adoption by at least 100 animal shelters [5]. The purpose of our study is to evaluate MYM^®^ Feline-ality™.

In ASPCA’s initial testing during program design phases, as well as in the subsequent modification study, the period following the implementation of the MYM^®^ Feline-ality™ program was characterized by an increase in cat adoption rates, decreased euthanasia rates, decreased return rates, and shortened length of stay in the shelter for cats [3,4]. Yet, not all of these improved outcomes were attributable to owners adopting cats that matched the indicated Feline-ality™, as 45% of pairings examined were not exact matches [3]. In a follow-up survey to the ASPCA 2007 beta testing, respondents indicated that the cats they had adopted largely behaved as they expected them to, based on the information they had obtained during the MYM^®^ process [3]. The ASPCA data were in alignment with previous research: accurate expectations for a new pet have been demonstrated as contributive to adoption success [3,4,6,7,8,9,10,11].

For both adopters and shelter staff alike, overall satisfaction with the MYM^®^ Feline-ality™ program was described as high during the ASPCA’s original beta testing [3]. Five shelters participated in the beta testing—the Animal Refuge League of Greater Portland; the Wisconsin Human Society in Milwaukee; the Hamilton-Burlington SPCA in Ontario, Canada; the Kansas Humane Society of Wichita; and the Humane Society of Boulder [3]. All five shelters were private, not-for-profit organizations; two were open-admission shelters while three were closed-admission (open-admission means that owners can surrender their animal at any time and for any reason; closed-admission means that the shelter limits which animals they are willing to accept). ASPCA pilot testing was completed in multiple phases using boarding facilities and shelters [3]. Two of the shelters used in the pilot testing were also used in beta testing (the Animal Refuge League of Greater Portland and the Hamilton-Burlington SPCA). The Humane Society of Boulder, one of the shelters used in the pilot testing, was also used in the 2015 modification study (open-admission) [3,4]. Data regarding satisfaction following the implementation of MYM^®^ Feline-ality™ in shelters outside of the 2007 ASPCA pilot and beta testing are not available in the scientific literature, nor is any further evidence that the program is associated with the favorable changes in adoption, euthanasia, returns, and length of stay reported. Information about the results of the adoption of this program in the wider animal sheltering system would help applied animal behaviorists make evidence-based decisions about the allocation of the scarce resources available in animal care, management, and welfare.

In order to evaluate the MYM^®^ Feline-ality™ program, and in collaboration with the Seattle Animal Shelter (SAS), we considered three aspects of program evaluations: needs assessment, outcome evaluation, and process evaluation. Needs assessment involves identifying gaps in current practices that might interfere with desired outcomes and providing suggestions of ways to ameliorate obstacles. The needs assessment relevant to, but independent of, this present study identified multiple areas needing improvement, including standardizing the assessment of cats, for which it was recommended to use the MYM^®^ Feline-ality™ program [12]. The needs assessment is discussed and an outcome evaluation (analysis of the effectiveness of a program) and a process evaluation (analysis of implementation) are examined herein.

To the best of our knowledge, the present study represents both the first outcome evaluation and the first process evaluation of the MYM^®^ Feline-ality™ program since its introduction into sheltering practice. Using data from a five-year period (2015–2019) and based on findings described in the available literature on the program, we expected to see a favorable change in adoption, euthanasia, return, and length of stay for cats at SAS following implementation of the program. As part of a retrospective process evaluation, we also examined errors made during the implementation of the program.

## 2. Materials and Methods

### 2.1. Study Site

Data were collected in partnership with the Seattle Animal Shelter (SAS), a large, urban, open-admission animal shelter located in King County, Washington, USA. The cats were generally housed in metal cages, some with one clear plastic wall so they were visible to visitors. During quarantine, or while meeting potential adopters, the cats may have been housed in other smaller rooms, though still most often singly. Cats were provided with litterboxes, bedding, and toys. Many cats were provided hiding areas. Dry cat food was available at all times and canned food was provided twice daily. Water was refreshed twice daily. Multiple trained staff members primarily carried out cat handling, cage cleaning, and feeding. Volunteers provided physical interaction to the cats, including brushing, petting, and playing. The study was reviewed and approved by the University of Washington Office of Animal Welfare (Protocol #2858-09).

### 2.2. Shelter Adoption Procedures

For the study period (2017), SAS used the ASPCA MYM^®^ adoption program and evaluated cats using the Feline-ality™ assessment protocol [3,4]. This program is designed to pair people with cats based on cat behavior and owner expectations. The MYM^®^ Feline-ality™ program comprises a behavioral assay administered to eligible cats and a survey completed by prospective owners. The behavioral assay is an 11-item test based on observing and recording a cat’s behavior in various situations (Table S1), and results in the assignment of one of nine possible “Feline-alities™” or cat personality types (Figure S1). Each of these Feline-alities™ is made up of scores along two personality dimensions, “Valiance” and “Independent/Gregarious”, which describe confidence and social attitude toward humans, respectively (Figure S2). The assignment of the specific Feline-ality™ is indicated by a colored card (purple, orange, or green) with a personality description placed on each cat’s cage. Prospective adopters complete the Cat Adoption Survey (CAS), in which they indicate their expectations of their ideal cat, as well as describe their lifestyle (Figure S3). Based on the score from the CAS, a person is provided with a compatible Feline-ality™ and given a colored card that matches the cards found on cages housing appropriate cats, as a reference tool when browsing available cats.

Accurate expectations of cat behavior during MYM^®^ are not obtained solely by the survey results and resulting cat personality description. In addition to the overall assignment of expected compatibility, it is assumed that the prospective owner will be led through the cat selection process by a shelter representative. The training materials support this assumption by providing advice on how best to talk to an owner about each item on the assay and how each should be interpreted in light of responses to the CAS—essentially, adoption counseling. This level of adoption counseling necessitates a high degree of training on the specific items in the assay and questions on the survey. It is not clear how shelter staff were trained to perform MYM^®^ during the ASPCA’s initial testing (though it may be safely assumed that they were trained by the researchers); currently, the ASPCA offers training at no monetary cost via online videos [13] and a 68-page training manual available for free download [14]. Along with conceptual training on the components of the program necessary to adequately counsel a potential adopter, shelter staff members also need training in the logistics of performing the assay, scoring the assay, and scoring the CAS. Each shelter is responsible for making sure that their staff and volunteers are trained sufficiently. There are no available data regarding shelter staff or adopter perception of the experience with implementation of the MYM^®^ program outside of the ASPCA pilot and beta testing [3].

SAS shelter staff were trained to direct the potential adopter towards the appropriate cats, as measured by the Feline-ality™ behavioral assay (Table S1; Figure S2). The animal control officers were responsible for all adoptions. Each of these six staff members, as well as one manager, received 14 h of training in 2016. Following the training, and after staff had time to practice the assay on shelter cats, each employee was required to accurately score a CAS and complete a Feline-ality™ assessment correctly. All training and evaluation were provided by the author (S.D.). The final evaluative Feline-ality™ assessment was completed in tandem with S.D. and, in order to be correct, the staff member must have categorized the test cat as the same Feline-ality™ as did S.D.

Staff training was conducted as an interactive discussion-based class, using materials provided by the ASPCA via their website [14], as well as earlier materials from the original CD-ROM version of the MYM^®^ Feline-ality™ program. Staff were trained to complete the modified Feline-ality™ assay [4], but some videos from the original assay [3] were still useful as additional practice opportunities during the training. In response to feedback from the staff, Supplementary Materials were created by S.D. (one document that outlined which behaviors each specific assay item should be associated with, and one document that condensed the general flow of the assay into one page for quick reference while practicing). SAS also requested a shelter-specific version of the training manual that delineated exactly how and where the assay should be done in their specific physical plant (this and all other training materials are available by request from S.D.). While staff members were the only people allowed to conduct adoptions, the shelter did have volunteers who interacted with potential adopters as they perused the available cats. Therefore, volunteers were also trained in the MYM^®^ program. Volunteer training did not focus on performing the behavioral assay, but rather on how the results of the assay, combined with the CAS, could best be interpreted and used as a tool for guiding potential adopters in their decision-making process. Two four-hour volunteer training classes took place in person with S.D. and, for other (or future) volunteers, a shelter-specific training video was created by S.D. and provided to the shelter, along with a quiz and self-test materials. Self-test materials for future training of staff members were also provided; training for any staff hired after the initial training provided by S.D. was completed by already-trained staff (this applied to one additional animal control officer who joined the team in March of 2017).

The decision to implement the ASPCA MYM^®^ protocol was made independently of this research, as a result of the broad needs assessment previously solicited by SAS and provided prior to the conception of this study. In the years 2015, 2016, 2018, and 2019, adoption procedures did not include MYM^®^ Feline-ality™. The initial needs assessment indicated that standardized adoption procedures were not in place prior to MYM^®^ [12]; information about adoption procedures subsequent to cessation of the MYM^®^ program were unavailable. This pattern of adoption procedures allowed us to compare our focal study year (2017) to the two years prior and the two years after implementation of MYM^®^ Feline-ality™.

### 2.3. Subjects

#### 2.3.1. Longitudinal Shelter Data (Outcome Evaluation)

Computerized records for 5 years (2015–2019) from the Seattle Animal Shelter (SAS) were examined to ascertain data regarding length of stay in the shelter, adoption rates, euthanasia rates, transfer rates, and return rates (following adoptions). Only cats that could have hypothetically benefited from the Feline-ality™ program were considered. Therefore, any animals whose age estimate was below 9 months of age or for which no age estimate existed were excluded from the analysis, as well as any cat for whom there was no outcome listed (missing data). All cats who died in care; arrived deceased; or were euthanized as a result of injury, illness, or other emergency less than 18 h after arrival at the shelter, as well as all cats labeled feral or wild, were also removed from consideration. Owned cats were excluded; that is, strays that were subsequently returned to the rightful owner; cats who entered legally required bite quarantine and subsequently returned home; all cats who visited the shelter only for services through the shelter-run veterinary clinic; and cats being held for safekeeping during domestic violence situations (returned to owner). Cats brought to the shelter solely for the purpose of humane euthanasia at the request of the owner were also excluded. It should be noted that the number of returned cats listed for each year does not equal the number of cats that had two visits within that calendar year; some cats were adopted in a different calendar year and then returned during the year in question—a difference that was often an arbitrary side effect of the cut-off dates chosen for analysis (such as adopted in December 2014 and returned in January 2015). Moreover, outcomes (adoptions, euthanasias, and transfers) were counted as unique events, rather than individual animals (some cats had two or more different outcomes), so the number of unique cats admitted each year was necessarily lower than the sum of the possible outcomes. Animals that remained following the guidelines above are described in Table 1.

A second sample of cats from another shelter located in Portland, Oregon was also collected (*N*_2015_ = 2609; *N*_2016_ = 2639; *N*_2017_ = 2767; *N*_2018_ = 2401; *N*_2019_ = 2581). The level of detail described (above) for our primary dataset was not available for the second dataset, which means that the outcomes (adoptions, euthanasias, and transfers) do contain an unknown number of kittens and feral cats and should be interpreted accordingly. Moreover, in the primary dataset, there were more outcomes than unique cats, which appears to differ from the comparison group (whose outcomes and number of cats total up to 100%).

#### 2.3.2. Feline-Ality™ Data (Process Evaluation)

Records were examined for 183 cats that completed the Feline-ality™ behavioral assay between 14 February 2017 and 31 December 2017 (34.7% of eligible cats taken in during that year). Of these, cats were 52.5% female and 98.3% altered (three females were unaltered or could not be verified as spayed either visually or via palpation of an abdominal scar). These cats ranged in age at intake from 9 months to 18 years (M = 6.5 y, SD = 4.5 y).

The Feline-ality™ behavioral assay consists of 11 test items that measure two correlated feline personality traits: Independence/Gregariousness (a measure of sociability) and Valiance (a measure of confidence/shyness achieved by recording responses to novel stimuli) [3,4]. The scores for each dimension range as follows: Independent/Gregarious ranges from two to thirty-two; Valiance ranges from zero to forty-three. Each dimension is subdivided into three categories that, when combined, form a matrix of nine possible Feline-alities™, each with their own description (Figures S1 and S2): Private Investigator, Secret Admirer, and Love Bug (low valiance cats, scoring 0–13); The Executive, Sidekick, and Personal Assistant (medium valiance cats, scoring 14–28); and MVP, Party Animal, and Leader of the Band (high valiance cats, scoring 29–43). The distribution of observed Feline-alities™ in this SAS sample was as follows: Private Investigator = 25%, Secret Admirer = 17.5%, and Love Bug = 0.0%; The Executive = 1.1%, Sidekick = 30.1%, and Personal Assistant = 20.8%; and MVP = 0.0%, Party Animal = 0.0%, and Leader of the Band = 5.5%. Training materials provided by the ASPCA describe the results of the original validation testing as having a “majority” of medium valiance cats, a “fair amount” of low and high valiance cats, and “few” Executives and MVPs [3], which seems to resemble our sample. The modification study cites the three most common Feline-alities™ as Personal Assistant, Leader of the Band, and Sidekick, which resembles our sample in that those three Feline-alities™ make up 56.4% of our sample, though we did not have many Leaders of the Band (*N* = 10).

### 2.4. Design

#### 2.4.1. Outcome Evaluation: Longitudinal Shelter Data and Variables

Retrospective statistical analyses of computerized records kept for each cat were performed, using a controlled interrupted time series (CITS) quasi-experimental design at the level of averages across years, including and comparing data from two years prior to the implementation of the MYM^®^ program (2015, 2016), the year in which the program was implemented (2017), and two years subsequent (2018, 2019). Ideally, in order to make inferences about the effect of Feline-ality™, a true experimental design with random assignment to treatment and control groups would be employed. Non-experimental designs are subject to threats to internal validity; that is to say, identifying whether the intervention in question actually caused any observed effect. Quasi-experimental designs are able to address some of these threats to internal validity to varying degrees. The main threats to internal validity, or inferring causality, are as follows: maturation, testing, instrumentation, regression to the mean, selection, history, and attrition [15,16,17]. For a detailed discussion of these threats as related to the current study, see the Supplementary Materials (Figure S5). While quasi-experimental designs do improve the ability to interpret the effect of the intervention on the sample, they should not be solely depended upon to infer that these effects, if any, would be repeatable in a different sample, or necessarily apply to a larger population. True statistical inference is not possible here; all data should be viewed as descriptive in nature and should inform any large decision only in concert with other work.

The appropriate way to control for many threats to internal validity in quasi-experimental designs is to include a non-equivalent control group for comparison. A non-equivalent control group is one that is similar to the treatment group but did not experience the treatment. The ideal non-equivalent control group in our study was a municipal shelter operating in a nearby city of similar size. Records from the Multnomah County Animal Shelter located in Portland, Oregon are publicly available and were our best option for a non-equivalent control group [18].

Variables for our non-equivalent control group were limited by available public records and included only cat adoptions, euthanasias, and transfers to another facility as outcome measures. Variables for our primary dataset (SAS) were defined as follows: *Length of stay*: the amount of time between admission to the shelter and being released from the shelter, calculated from the exact date and time that each cat was entered into the computer by staff as having arrived and left the shelter; *Outcome*: adoption, euthanasia, or transfer to another facility; *Returned cats*: cats recorded as returned in the database; any cat that had been previously adopted from this shelter and then surrendered back to this shelter within each calendar year from 2015 through 2019 (though not always marked as returns in the database), as well as cats that were recorded as returned but had been adopted previously in a different calendar year. It should be noted that the number of cats recorded as returned was always less than the number of cats that actually had more than one surrender (failed adoption) to the shelter per each calendar year, pointing to a problem in data accuracy at the shelter level. Because of these discrepancies, it is likely that return estimates in the SAS sample are conservative.

#### 2.4.2. Process Evaluation: Feline-Ality™ Data and Variables

The ASPCA MYM^®^ program is composed of two parts, the Feline-ality™ behavioral assay and the Cat Adoption Survey (CAS). The behavioral assay is composed of 11 items, described in detail in the ASPCA training manual and available online [3,4,14]. See Table S1 for a description of the assay, as well as scoring information; Figure S2 for the chart used to translate the final scores along the two dimensions into specific Feline-alities™; and Figure S1 for descriptions of each Feline-ality™. The CAS is provided in Figure S3. Examined variables were composed of possible implementation errors.

Possible errors in the Feline-ality™ assay included the following: (a) mathematical errors in which negative numbers were incorrectly added or subtracted, resulting in an incorrect score for the category; (b) errors when transferring individual item scores to the last page where scores were finally summed; and/or (c) mathematical errors in summing the scores in individual items and in the final summation. All of these errors have the potential to affect the assigned Feline-ality™.

Possible errors in the Cat Adoption Survey (CAS) included the following: (a) incomplete owner information—failure to answer any question on the survey renders it unscorable. This issue came up especially with question 6, regarding children in the home (a fourth option), “Children do not often come to my home” is offered in a column that is not counted for scoring and, once selected, seemed to lead people to not select one of the other three options that are required for scoring (see Figure S3); (b) staff error—these often included math errors, especially with question 6 (which needs to be used twice in the calculations) and question 1, which was often left out altogether; (c) failure to recommend the correct Feline-ality™ to a potential owner, even with correct math; (d) failure to record an assigned Feline-ality™ despite correct and complete owner information; and (e) failure to complete a CAS at all.

### 2.5. Statistical Tests

Changes in the outcome variable Length of Stay across years (a continuous variable) were examined using one-way analysis of variance. Changes in other outcomes (adoption, euthanasia, transfer, and returns) were examined using chi-square tests of independence (frequency data). We analyzed return rates separately from adoption, euthanasia, and transfers, as returns were an outcome that only applied to animals that were adopted, a subset of our sample. Significance levels were set to *α* = 0.05, and Cramer’s v was calculated for the effect size of chi-square tests [19,20]. The interpretation of Cramer’s v for contingency tables with more than two categorical variables depends on the chi-sq degrees of freedom; our results should thus be interpreted using the following effect size heuristic: small > 0.035, medium > 0.11, and large > 0.18 [19,20]. No statistical comparisons to or inferences about were or can be made about the broader population of shelter cats in general using the Feline-ality™ data; these results are descriptive only.

## 3. Results

### 3.1. Longitudinal Shelter Data (Outcome Evaluation)

#### 3.1.1. Primary Shelter Data (SAS)

**Figure 1:**
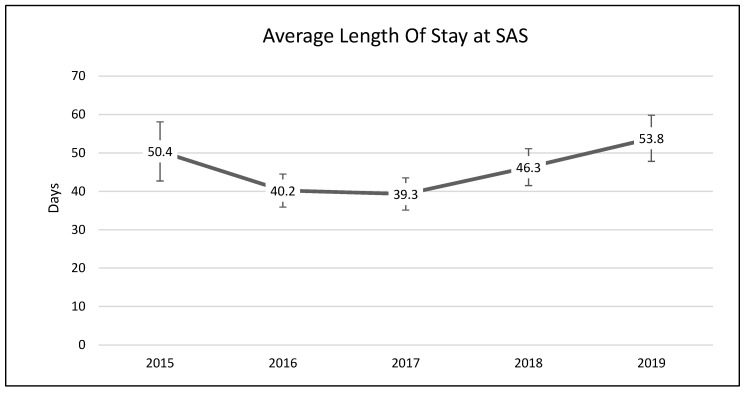
The average length of stay (LOS) for adult cats across a five-year period at SAS. The Meet Your Match^®^ program was implemented in 2017. Error bars represent standard error.

**Figure 2:**
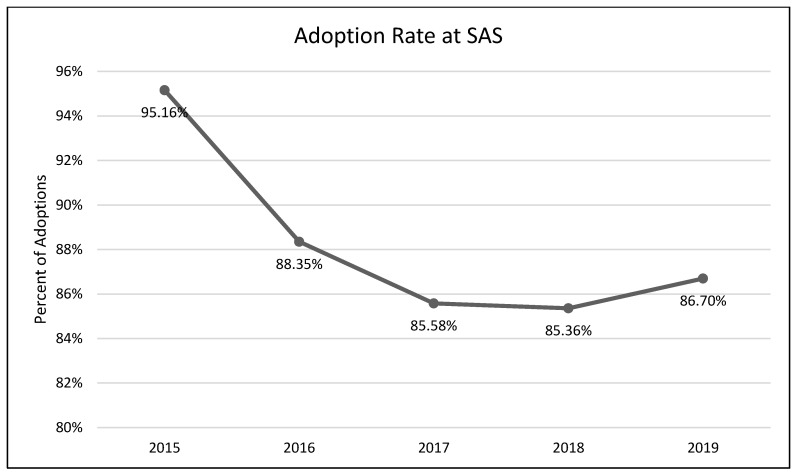
The percentage of adult cat adoptions across the five-year period (SAS).

**Figure 3:**
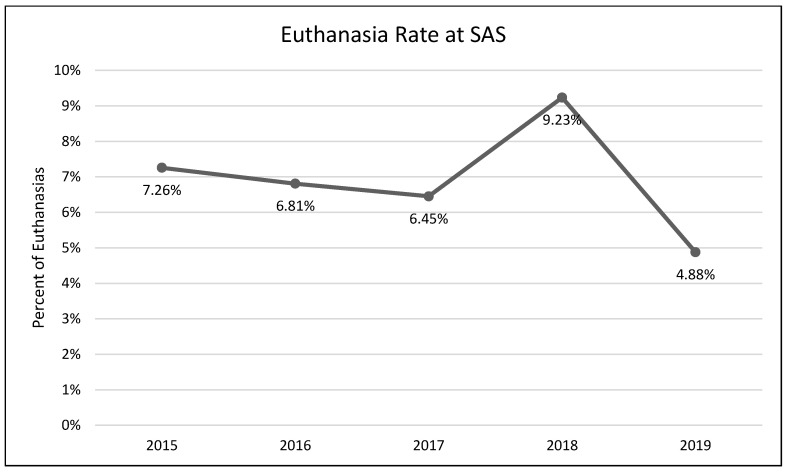
The percentage of adult cat euthanasias across the five-year period (SAS).

**Figure 4:**
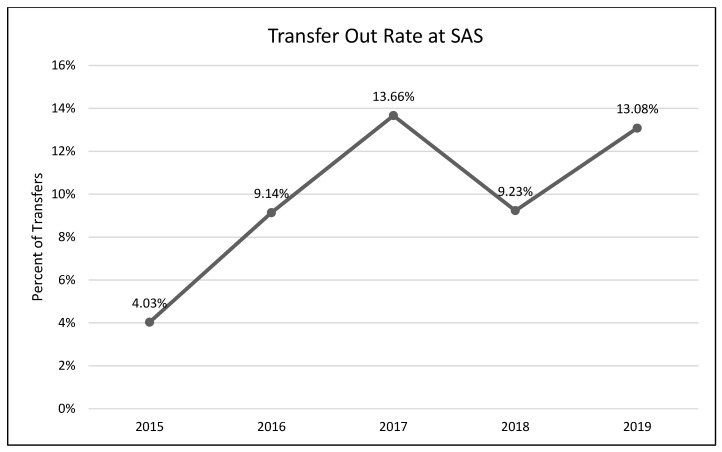
The percentage of adult cat transfers to other sheltering facilities and organizations during the five-year period (SAS).

**Figure 5:**
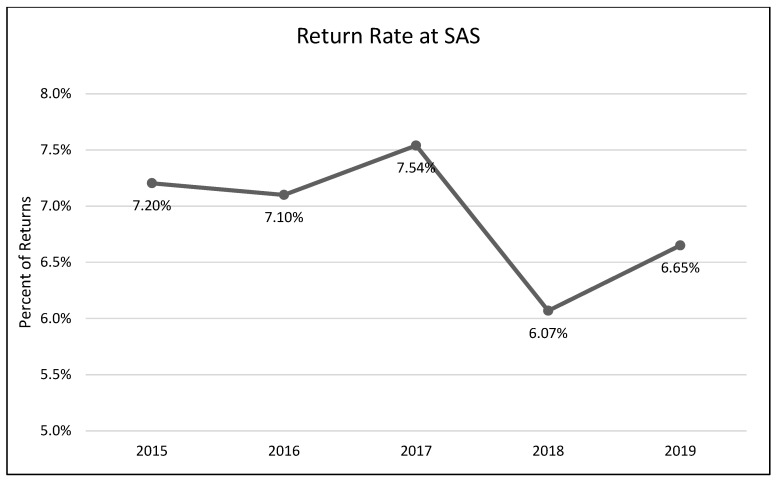
The percentage of adopted adult cats returned to the shelter after adoption across the five-year period (SAS).

A one-way analysis of variance showed no significant differences between average length of stay across the years 2015–2019 (*F*(3, 2331) = 1.54, *p* = 0.19; Figure 1). A chi-square test of independence was performed to examine the possible relationship between year and distributed frequency of outcomes (adoption, euthanasia, or transfer to another facility). The relationship between year and outcome pattern was significant and small (χ^2^(8, *N* = 2336) = 27.79, *p* < 0.001, *v* = 0.08). This significant overall χ^2^ appears to have been driven by lower than expected transfers in 2015, higher than expected transfers in 2017, higher than expected euthanasia rates in 2018, and lower rates of euthanasia and higher rates of transfer in 2019 (Table 2; Figure 2, Figure 3 and Figure 4). A chi-square test of independence was also performed to examine the relationship between year and frequency of returned adoptions. The relationship between these two variables was not significant (χ^2^(4, *N* = 1950) = 0.79, *p* = 0.94; Figure 5).

#### 3.1.2. Non-Equivalent Comparison Shelter (MCAS)

**Figure 6:**
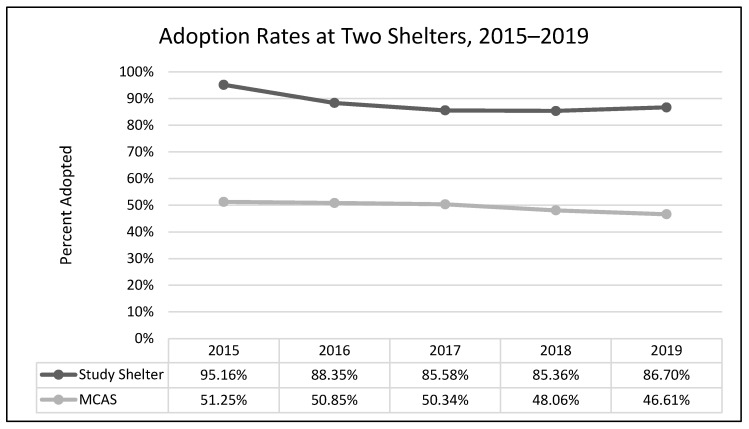
The percentage of adult cat adoptions across the five-year period at two shelters.

**Figure 7:**
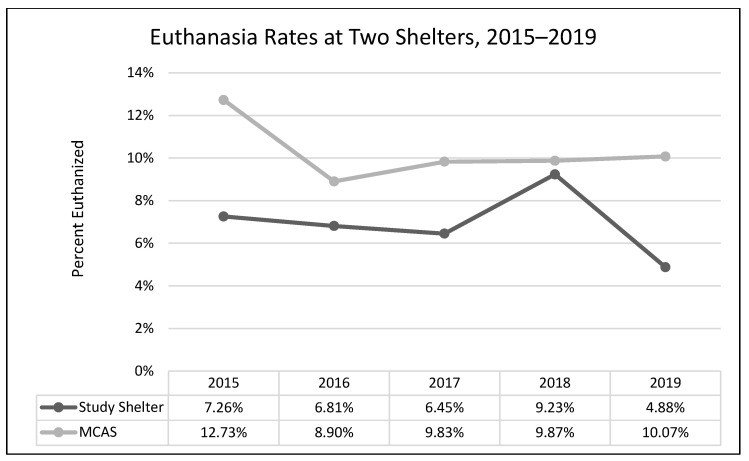
The percentage of adult cat euthanasias across the five-year period in two shelters.

**Figure 8:**
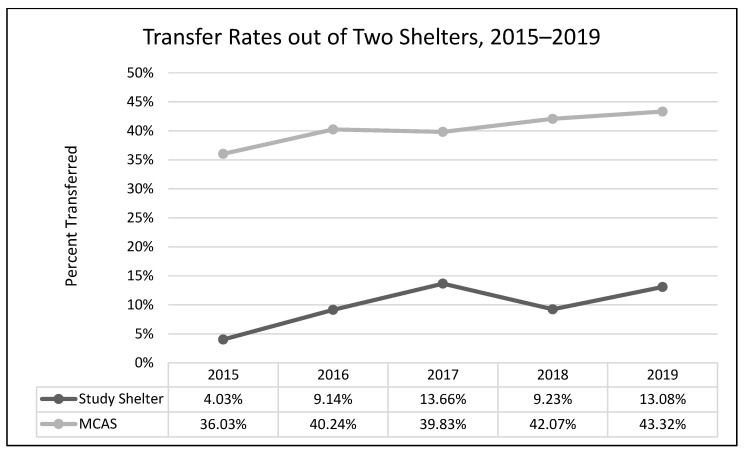
The percentage of adult cat transfers to other sheltering facilities and organizations during the five-year period at two shelters.

The relationship between year and outcome was significant and small (χ^2^(8, N = 12,997) = 49.08, *p* < 0.001, *v* = 0.04). The significant χ^2^ appears to have been driven by higher than expected rates of euthanasia and lower than expected rates of transfer in 2015, followed by lower than expected rates of euthanasia in 2016, and lower than expected rates of adoption and higher than expected rates of transfers in 2019 (Table 3; Figure 6, Figure 7 and Figure 8).

#### 3.1.3. Feline-Ality™ Data (Process Evaluation)

Errors in calculating the results of the Feline-ality™ behavioral assay created small but important mathematical differences that resulted in some cats being assigned the incorrect Feline-ality™. Out of 183 Feline-ality™ assessments available for review from 2017, 26 (14.2%) had errors, 8 of which resulted in the incorrect Feline-ality™ being assigned. Of the 183 adoptions in 2017 in which Feline-ality™ assessments were available for review, 102 had no Cat Adoption Survey (CAS). Of the remaining 81 CASs, 68 (84%) had one or more errors, with the most common being incomplete owner information (78%).

Of the 183 cats, 129 had either no CAS or no Feline-ality™ recommendation recorded as having been suggested to their adoptive owner, so it is impossible to say if those cats were correctly matched using the MYM^®^ system. Of those for whom recommendations were made (54), 9 cats were correctly matched with adopters on both IG and Valiance, 3 cats were matched correctly on Valiance only, and 42 cats were not matched as indicated by the test and survey results. Overall, a total of 3 cats were matched correctly with no errors in either the CAS or Feline-ality™, a 1.6% overall success rate.

## 4. Discussion

The outcome evaluation presented herein, when viewed in tandem with the process evaluation that revealed an extremely high error rate, provided no evidence of any effect of the MYM^®^ Feline-ality™ program at our study shelter.

The MYM^®^ program at Seattle Animal Shelter (SAS) was begun in early 2017 and continued through the end of that year. Outcomes were examined for 2 years prior to Feline-ality™, the year of its implementation, and two years subsequent (2015–2019). Length of stay was predicted to decrease after Feline-ality™ began (2017); length of stay did not significantly change across years. The return rate was also predicted to decrease; the return rate did not change significantly across years. Adoption was expected to increase in 2017, and euthanasia and returns were expected to decrease in 2017; we had no prediction regarding the transfer rate as Feline-ality™ makes no claims regarding an effect on transfers. We also had no specific predictions for 2018 or 2019. The distribution of outcomes (frequency of adoption, euthanasia, and transfer to other facilities) varied significantly across the across the five-year study period at SAS. However, it is important to note that the chi-square test is a global test and does not identify which specific outcome drove the overall significance. The significant chi-square result might have been driven by any of the changes we see in the visual representation of the data. However, this is a descriptive interpretation and does not imply inferential or generalizable results. The overall adoption rate at SAS fell across the 5 years, as did euthanasia rates. The changes in adoption rate and euthanasia rate in 2017 were negligible (and running counter to our prediction), followed by an increase in euthanasia in 2018. The transfer rates out of SAS increased overall and the changes year-over-year in transfer rates appear to have contributed the most to the overall significant chi-square. The euthanasia rate initially was trending downward across 2015 through 2017, spiked upward in 2018, and appeared to be decreasing again in 2019. The SAS euthanasia rate pattern seems to be the opposite of the transfer out rate pattern: as more cats are transferred to other facilities, the need for euthanasia may decrease (and vice versa). This inverse correlation between euthanasia and transfer is sufficient to explain the overall significant differences seen in our data. If there was any effect of MYM^®^ in 2017, this effect does not appear to have persisted beyond that year, not unsurprisingly given the extremely small effect size describing the differences in outcome variables between years. These small and short-lived effects seem to indicate either that the MYM^®^ Feline-ality™ program is an ineffective program and/or that it was poorly implemented.

Overall, transfer rates increased in both shelters (SAS and MCAS), and adoption and euthanasia rates decreased (no data for return rates or length of stay were available from the comparison shelter). The apparent spike at our study shelter (SAS) in euthanasia in 2018, mirrored by the 2018 dip in transfer rates, is the only deviation from the similarity in overall patterns between the two shelters. However, when compared with publicly available data on overall trends in SAS transfers and “other than live releases” (which include euthanasia and other deaths) for our study shelter (2016–2019), this same mirrored spike occurred shelter-wide (including all species handled by SAS) and is thus not limited to cats, nor attributable to Feline-ality™ [21]. Furthermore, the similarity in trends across years for the two shelters fails to rule out the possibility that history or maturation effects occurred at SAS; it is possible and even likely that the trends in both shelters were due to some alternative explanation(s), the most likely of which might be overall changes in shelter practices and attitudes over that time period. Since the Asilomar Accords of 2004, during which a group of 20 animal sheltering stakeholders (including the ASPCA) called for a national database to track sheltered animals and their outcomes [21], there has been a concerted effort and some success in the animal sheltering community at large to increase the live release rate and decrease euthanasia, often by transferring animals taken in by large or municipal shelters to other animal rescue organizations [22]. This explanation explains our data better than MYM^®^ Feline-ality™. See Figure S5 for a thorough exploration of systematic alternative explanations addressed by our study design.

Program evaluation is composed of multiple components, the first of which is a needs assessment. In the needs assessment commissioned by the shelter, SAS was advised regarding multiple areas that could be changed in order to improve overall adoption success and well-being. For cats specifically, aside from the MYM^®^ Feline-ality™ program, it was also suggested that the shelter should improve training of volunteers and staff in basic feline maintenance and behavior, provide enrichment to cats (e.g., olfactory stimulation, food puzzles, and auditory enrichment), and change and codify cage housing procedures for cats (e.g., provide hiding areas, limit exposure to dogs, place outgoing cats in more exposed cages, while reserving quiet cages for new or scared cats). Given our quasi-experimental design, it is important to consider what effect, if any, the needs assessment might have had on our results.

In terms of the needs assessment, any effect caused by its existence and dissemination, or changes made as a result of its suggestions (outside of the MYM^®^), would fall under the category of historical, or external, threats to inferring causality. Simply being exposed to the needs assessment, prior to any further intervention, could induce behavioral changes in shelter staff that might improve outcomes for cats, as staff attempt to ameliorate problems that they have now become aware of (e.g., seemingly aggressive cats transferred out rather than euthanized), or might alter the outcome data in other ways, such as lowering the average length of stay, as more cats are transferred out. The needs assessment was conducted in early 2016 and shared with the shelter in May of 2016; transfer rates did indeed begin to increase in 2016 and continued to do so in 2017. However, if there was an effect of the needs assessment that drove transfer rates initially, that effect disappeared by 2018 and thus cannot explain the rebound in 2019. Given that transfer was not specifically mentioned as a suggestion in the needs assessment, we consider any effect of the assessment on transfer rates to have been unlikely to induce any lasting change in cat outcomes.

Specific suggestions made in the assessment might also pose history threats to the internal validity of our outcome evaluation. Improving training for staff and volunteers regarding cats overall is one such possibility. As staff became more aware of species-specific considerations and behavioral indicators related to which would benefit from a foster home, which might benefit from transfer to another facility, and which were likely to do well in a shelter environment, it is logical to assume that outcomes could be influenced. However, outside of the intensive Feline-ality™ training provided by S.D., there is little evidence that other training and education was undertaken at an appreciable scale. The SAS progress report summarizing the behavior program in May 2018 indicated that written volunteer materials were updated, and that four volunteers and two staff members (comprising the Cat Behavior Team) met with another area shelter with a well-established cat behavior program and received training in learning theory [23]. The Cat Behavior Team focused largely on cat behavior modification but, per the progress report, the need for such behavioral interventions decreased after changes in housing and enrichment, and the Cat Behavior Team was not expanded [23]. It is unknown how much of an effect this additional training might have had, but given the small number of people involved, it is reasonable to assume that it is unlikely to explain our results. Based on personal observation, many of the other recommendations regarding enrichment, housing, and exposure to dogs were either not instituted or instituted haphazardly or inconsistently, and therefore were unlikely to have influenced our results in a systematic fashion.

Another alternative explanation, suggested by a staff member in an internal program evaluation submitted to management at the end of 2017, attributed falling euthanasia rates in 2017 to a hospice care program (“Fospice”), instituted in 2016, in which cats with a diagnosed illness and a prognosis of less than six months were placed in long-term palliative foster care, rather than euthanized [24]. However, while Fospice does indeed delay euthanasia, it does not prevent it, and this delay in euthanasia does not do a better job of explaining the subsequent pattern we saw in euthanasia rates in 2018 (spike) and 2019 (drop), than does the change in transfer rates. It is more likely that the Fospice program would have affected the average length of stay for cats, as animals that would have previously been euthanized owing to terminal illness instead lived longer in foster care. However, LOS did not change significantly over the study period.

The same 2017 internal staff evaluation also suggested that there might have been an effect on cat outcomes related to the hiring of full-time veterinary staff and/or the creation of a cat adoption follow-up team [24]. However, it reasonably follows that an increase in diagnosis and in-shelter treatment of manageable medical conditions would have increased the pool of cats that would now be deemed adoptable, and that the diagnosis of unmanageable and terminal illnesses would either increase the euthanasia rate or increase the number of cats eligible for Fospice (which, if happening at a high rate, should have showed up in our LOS data), none of which explain the changes in adoption rates or euthanasia rates in our data, either in 2017 or in years following. Return rates did not change significantly, providing no evidence that the cat adoption follow-up team was a significant factor in our findings.

Whereas the lack of substantive effect may indicate that MYM^®^ Feline-ality™ does not perform as purported, it is equally as likely (if not more so) that this absence of evidence of any results reasonably attributable to Feline-ality™ has occurred because the error rate in implementation of the program was very high. The retrospective process evaluation revealed that only three cats were matched with their adopters along both Feline-ality™ dimensions without any errors in either the assay or the survey. Nine cats were matched correctly along both dimensions despite errors in the underlying paperwork. In the original work creating the MYM^®^ Feline-ality™ program, the ASPCA describes that approximately 45% of cats were matched perfectly to their new owners, as compared with 4.9% at our primary study shelter [3]. Furthermore, the majority of cats were paired without any record of the adoptive owner having completed a CAS. Of those that did complete the CAS, the error rate was 85%. The Feline-ality™ behavioral assay also showed errors. However, the assay could more flexibly absorb errors owing to the range of numbers allowable by the scales; errors caused a cat to be incorrectly labeled as the wrong Feline-ality™ in only 4.4% of the cases. The fact that a poorly implemented program necessarily impacts the results of an outcome evaluation underscores the need for process evaluation concurrent with implementation.

The lack of effect may have occurred because the error rate in implementation of the program was very high. It is also possible that the program is fundamentally flawed in some other way that renders improvements in implementation moot; see [25] for an exploration of the underlying personality trait measurement that forms that basis of Feline-ality™. It is also possible that our study shelter may have differed in other ways that could have directly contributed to the poor implementation of the MYM^®^ Feline-ality™ program. Specifically, the study shelter suffered from inadequate staffing levels and an inefficient distribution of labor [12]. It may also have occurred because SAS stopped implementing the MYM^®^ program as it was designed. In early 2018, the shelter reported that it had modified the program such that most cats were “fast-tracked” and only a subset of less well-socialized cats were given the entire Feline-ality™ assessment. Fast-tracking was described as follows in an internal staff report.

“Once the cat has eaten on its own, been in the shelter a minimum of 18 h, and is medically sound enough to go through a Feline-ality™ assessment, the assessment can start. The Assessment will start as any normal Feline-ality™ would, however if the cat has an aggregate score of 5 or higher on items #1 through #4, the cat will be fast tracked. If the aggregate score is below a 5, the remainder of the Feline-ality™ will be performed and the cat routed accordingly… Likely we would only be performing a completed Feline-ality™ on what would be deemed purple cats (Private Investigator/Secret Admirer/Love bug), which are also the harder adoption cases aside from cats that are medically fragile or have more profound behavior concerns (forwardly aggressive). We would still keep the application process the same and there would be no changes done to the “cat adoption survey” at this time” [24].

Fast-tracking may have started earlier than management was made aware of. There also may have been attrition in completion rates (another threat to internal validity). Either could account for the fact that only 183 cats were recorded as having undergone the Feline-ality™ testing in 2017, despite the fact that 527 cats that theoretically could have been assessed were taken in during 2017. Fast-tracking could also explain why our distribution of Feline-alities™ is 42.5% “purple” or lower valiance cats, and only 5.5% are “green” or high valiance cats (it is impossible to assess whether our distribution is statistically significantly different from previous distributions, as the distributions of Feline-alities™ in the ASPCA training manual and Weiss et al. were not described in detail [3,4].

SAS suspended Feline-ality™ assessments between January 2018 and April 2018 in order to evaluate whether length of stay (LOS) would be affected. According to their internal analysis, average LOS was not affected by the suspension of the assessment and remained stable, though variability appeared to decrease [23]. The shelter interpreted the results of their evaluation to indicate that the MYM^®^ program was not delivering the desired outcomes, but that perhaps simply training staff to better recognize body language and understand feline behavior was improving overall cat adoption decisions [23]. It was their final conclusion that they should therefore “continue to train Animal Care staff on a formal program to recognize cat body language and behavior that leads to good routing decisions” [23]. However, the technique used to evaluate the change in LOS and the change in variability in LOS was not statistically rigorous. Our post-hoc analysis established homogeneity of variance before and after the implementation of the MYM^®^ Feline-ality™ program, rather than the decreased variability suggested in the report (S4). The extreme decrease in LOS in February of 2017 is likely attributable to the statistical phenomena of regression to the mean, and does not represent an effect of Feline-ality™, nor can the variability be interpreted as evidence of an effect of general cat behavioral training, as was suggested in the SAS report [23].

At this point, it is unclear whether any form of the MYM^®^ program remained in place after January of 2018, nor is it clear what, if any, formal cat behavior training continued after this date. All of these things combined with poor implementation of the program may explain the falling adoption rates, rising return rates, and increasing lengths of stay that appeared to be recurring by the end of the study period. It is, however, both impossible to attribute any changes to MYM^®^ Feline-ality™ and equally as impossible to rule out any effect; without proper implementation, an outcome evaluation is uninterpretable.

Our work has revealed some weaknesses inherent in the MYM^®^ Feline-ality™ program that contribute to difficulties in implementation. First and foremost, the fact that the CAS allows for no human error is simply not practicable. Any of the first ten questions not answered or answered incorrectly, and not caught and corrected by the staff, can and did lead to errors that prevented accurate matching between prospective adopters and appropriate cats. A difference in just one point in scoring the CAS can lead to assignment of the incorrect Feline-ality™ match, which shows that the behavioral assay is more robust to human error because of the larger range of values that describe the Feline-alities™; small math errors are less likely to have an effect. It is not reasonable to assume that staff will catch all errors on the CAS and some error rate in the overall program is to be expected. However, the high error rate seen here may, at least in part, indicate that the training materials provided by the ASPCA are inadequate.

The materials available for training in the MYM^®^ Feline-ality™ program are lacking in some key areas. There is one video for practicing the entire behavioral assay and one portion of a video that walks through the scoring of the CAS. Because we had access to the DVD from the original program, we were able to piece together an additional video of the full assay for use as practice in the training sessions, as well as two additional CAS practice videos. As part of their training, staff also practiced the full assessment on live cats during training, with S.D. there to provide feedback, as well as practiced scoring new CASs provided by S.D., with oversight from S.D. Furthermore, the CASs provided by the ASPCA for scoring practice did not include any errors, so learners would not have the opportunity to practice catching errors; in our training, this was provided. Additionally, the ASCPA materials do not provide any other opportunities for self-evaluation, such as quizzes; we created quizzes and incorporated them into both staff and volunteer training. All of this means that our study shelter actually had more extensive training on the assay and the CAS than they would if they had only used the ASCPA materials, though by necessity, on an ad hoc basis. There is no information that describes how other shelters might use or improve upon the materials provided by the ASPCA; there is likely to be variability in this aspect of the program implementation, which points to weaknesses in elements that are necessary prior to the successful adoption of a new program, namely diffusion, and specifically dissemination and replication (for a useful definition of these terms, see Goldman [26]).

Another potential area of weakness is in the training surrounding adoption counseling. As far as we are aware, there are no data regarding the efficacy of this aspect of the online training (communicating the results of the Feline-ality™ assay and the CAS effectively to potential adopters). The focus of this part of the training material is on presenting the information to adopters using specific soft skills (such as keeping one’s tone positive or ascending, using open-ended questions, paraphrasing to indicate listening, and so on). Soft skills are notoriously difficult to train and even more so via non-interactive online media [27,28]. During training at our study shelter, we did roleplay adoption counseling and it is possible that other shelters might do this, too. Based solely on the materials from the ASCPA, however, it is unlikely that adoption counseling techniques would be affected, which could also adversely affect the implementation of the MYM^®^ Feline-ality™ program.

## 5. Conclusions

To the best of our knowledge, this study represents the first systematic program evaluation of MYM^®^ Feline-ality™. The results of our evaluation indicate that successful implementation of the program requires proper consideration of the process of implementation, ideally both concurrent with and subsequent to implementation, and that without attention to that process, the error rate in implementation may render any outcome evaluation problematic, as it did here. From an applied science perspective, this is concerning. If funding allocation decisions are made based on an outcome evaluation that is in turn based on poor implementation, the risk of either rejecting useful programs or continuing to fund useless programs is unacceptably high.

## Figures and Tables

**Table 1:** Summary subject and descriptive data, SAS.

Year	Admissions	Adoptions	Euthanasias	Transfers	Returns	Age	Average Length of Stay	Sex
2015	248; 16	236	18	10	17	Range: 3 months to 17 years (M = 5.85 y, SD = 4 y)	1.7 months (M = 50.4 d, SE = 7.9 d)	53% male; 99.2% altered
2016	558; 24	493	38	51	35	Range: 9 months to 20 years (M = 5.65 y, SD = 3.9 y)	1.3 months (M = 40.2 d, SE = 4.3 d)	54% female; 0.01% unknown; 95.5% altered
2017	527; 27	451	34	72	34	Range: 9 months to 28 years (M = 5.55 y, SD = 4.2 y)	1.3 months (M = 39.3 d, SE = 4.2 d)	56% female; 0.01% unknown; 96.5% altered
2018	444; 15	379	41	41	23	Range: 9 months to 18 years (M = 6.15 y, SD = 4.75 y)	1.5 months (M = 46.3 d, SE = 4.8 d)	54% female; 0.001% unknown; 95.5% altered
2019	451; 21	391	22	59	26	Range: 9 months to 20 years (M = 6.5 y, SD = 4.75 y)	1.8 months (M = 53.8 d, SE = 6 d)	53% female; 0.004% unknown; 97.1% altered

The “Admissions” column shows the number of unique cats who fulfilled criteria that were admitted in each year, as well as the number of those cats that had two or more visits to the shelter during that same year. The “Sex” column also reports the percentage of animals that were verified to be altered. Some cats were unaltered or could not be verified as spayed either visually or via palpation of an abdominal scar; it is a reasonable assumption that all animals were desexed before being adopted, as per shelter policy.

**Table 2:** Frequency table for the primary shelter (SAS). Observed (expected) [contribution to overall χ^2^].

Study Shelter	2015	2016	2017	2018	2019
**Adoption**	236 (220.38) [1.11]	493 (485.83) [0.11]	451 (464.96) [0.42]	379 (384.82) [0.09]	391 (394.01) [0.02]
**Euthanasia**	18 (17.29) [0.03]	38 (38.12) [0.00]	34 (36.48) [0.17]	41 (30.19) [3.87]	22 (30.91) [2.57]
**Transfer Out**	10 (26.33) [10.13]	51 (58.05) [0.86]	72 (55.56) [4.87]	41 (45.98) [0.54]	59 (47.08) [3.02]

**Table 3:** Frequency table for MCAS. Observed (expected) [contribution to overall χ^2^].

MCAS	2015	2016	2017	2018	2019
**Adoption**	1337 (1290.55) [1.67]	1342 (1305.38) [1.03]	1393 (1368.70) [0.43]	1154 (1187.66) [0.95]	1203 (1276.70) [4.25]
**Euthanasia**	332 (268.19) [15.18]	235 (271.27) [4.85]	272 (284.43) [0.54]	237 (246.81) [0.39]	260 (265.31) [0.11]
**Transfer Out**	940 (1050.26) [11.58]	1062 (1062.34) [0.00]	1102 (1113.87) [0.13]	1010 (966.53) [1.95]	1118 (1038.99) [6.01]

## Data Availability

The data presented in this study will be made available in ScholarsArchive@OSU at https://doi.org/10.7267/9k41zp24k or upon request.

## Supplementary Materials

The following supporting information can be downloaded at https://www.mdpi.com/article/10.3390/ani13172752/s1. Table S1. The Meet Your Match^®^ Feline-ality™ behavioral assay including the final page of the Feline-ality™ assessment where scores on the 11 items are summed and a feline-ality is assigned [2,3]. Items 1, 2, 4, 5b, 6, 9, 10, and 11 are summed, and items 3, 5a, 6, 7, 8, and 9 are summed—note that items 6 and 9 are used in both summations. See ASPCA [3] for step-by-step details on the procedure for conducting and scoring the assay. Adapted from the ASPCA Meet Your Match^®^ Feline-ality™ Manual and Training Guide [2]. Figure S1. Descriptions of the nine possible Feline-alities™. Image adapted from the ASPCA Meet Your Match^®^ Feline-ality™ Manual and Training Guide [3]. Figure S2. The sum of items 1, 2, 4, 5b, 6, 9, 10, and 11 provides a total Independent/Gregarious (IG) score that is then located along the top of the chart and corresponds to the level of sociability that the cat is likely to show. Items 3, 5a, 6, 7, 8, and 9 are summed and compared to the chart on the left side, indicating the level of valiance (or confidence) a given cat is likely to demonstrate. Image adapted from the ASPCA Meet Your Match^®^ Feline-ality™ Manual and Training Guide [3,4]. Figure S3. Items 1 through 9 are required in order to accurately score the CAS and item 6 must be counted twice (the sum of 1–6, and then the sum of 6–9) for each column. Items 10 through 16 are for information purposes only. Questions in the fourth column, regardless of item number, are not included in the final calculations. The suggested matching Feline-ality™ is recorded on the bottom and owners are directed towards those cats whose behavioral assay result corresponds to their CAS result. See ASPCA [3] for a detailed description of scoring. Image adapted from the ASPCA Meet Your Match^®^ Feline-ality™ Manual and Training Guide [3]. Figure S4. Graph adapted from SAS in-house outcome evaluation [21], used to examine changes in LOS. * = 6 months of age and older. Explanation regarding statistical choices. Figure S5. Explanation of threats to internal validity addressed by study design [29].
