# Supplementary material for: Feline-Ality™ in Real Life: A Program Evaluation
Source: Animals (Basel). 2023 Aug 29;13(17):2752. doi: 10.3390/ani13172752 (PMC10486580; doi:10.3390/ani13172752)
Supplement: Supplementary file 1 [file animals-13-02752-s001.zip › Figure S1.pdf]

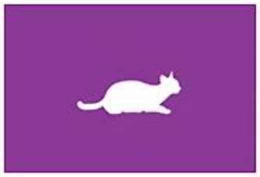

Private Investigator

I'm working undercover to keep an eye on you and your household. You may not even know you're under surveillance. I can vanish into thin air if anyone or anything interferes with my investigation. If you need a cat who knows how to stay out of trouble and will always keep your secrets, I just might take your case.

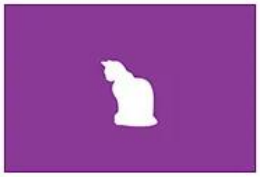

Secret Admirer

When it comes to relationships, I'm very level-headed. I don't leap in paws first, if you know what I mean. But give me a little time, and then I'll shower you with purrs, head-butts, and plenty of lap time. In the meantime, you may not see a lot of me, but I'll be thinking a lot of you!

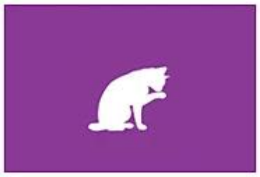

Love Bug

Do you seek affection? I do! If you also like petting, purrs, and paws kneading your lap, I think we might have a LOT in common. I'm looking for "someone who enjoys quiet times and togetherness." Could that someone be you?

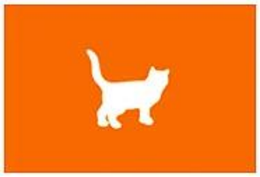

The Executive

I have to say, I'm a busy cat. First, I've got to check out what's happening out the window. Next, I'll see if any closets or cupboards need looking into. And then there are my naps--can't be late for those. I can fit a little socializing into my schedule. Shall we plan on breakfast and dinner? I hope you like kibbles.

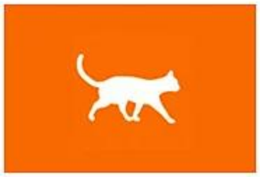

Sidekick

Like all sidekicks, I'm just plain good company. I like attention, and I also like my solitude. I don't go looking for trouble, but I'm no scaredy-cat, either. If you are looking for a steady companion to travel with you on the road of life, look no further.

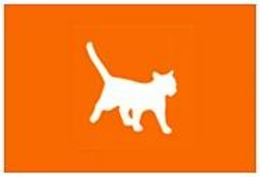

Personal Assistant

You're working on the computer? Let me press the keys. Reading the paper? I'll hold the pages down for you. Watching TV? I'll just plop in your lap so you can pet me. I love an orderly household, don't you? I'll help you with all your chores, and I'll help you relax when we're done. You'll wonder how you ever managed without me.

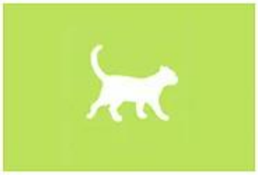

MVP

I'm a savvy cat who knows the score. I'm pretty unflappable, too. I don't mind entertaining myself, but a human companion at the other end of the couch and a nice scratch behind the ears always make my day. If you're looking for a resourceful addition to your team, think about signing this Most Valuable Pussycat.

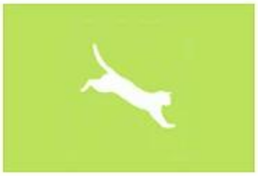

Party Animal

I'm a cat on a mission: PARTY! I love to play and explore and test my limits. I'd love to play with you, but I can make a toy out of anything: pencils, post-it notes, potatoes. If you're looking for some laughs and someone to liven up the party, think about inviting me.

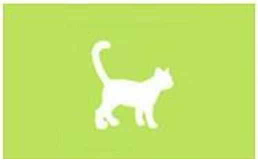

Leader of the Band

I'm a cat who does everything in a big way. I not only like to be in the middle of things--I like to lead the parade. I'm an adventurous cat, but I'll still make plenty of time to show you my affectionate side. I'm the demonstrative type, you might say. Want a cat who's brimming with confidence? That's me.
