# Supplementary material for: Feline-Ality™ in Real Life: A Program Evaluation
Source: Animals (Basel). 2023 Aug 29;13(17):2752. doi: 10.3390/ani13172752 (PMC10486580; doi:10.3390/ani13172752)
Supplement: Supplementary file 1 [file animals-13-02752-s001.zip › Figure S2.pdf]

# meet the feline-alities

I N D E P E N D E N T - G R E G A R I O U S

V A L I A N C E

| | independent (2–10) | social (11–20) | gregarious (21–32) |
|---|---|---|---|
| low valiance (0–13) | 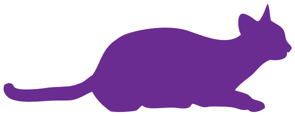 private investigator | 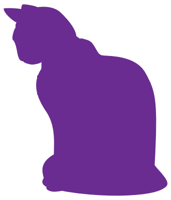 secret admirer | 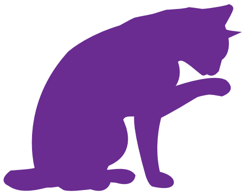 love bug |
| medium valiance (14–28) | 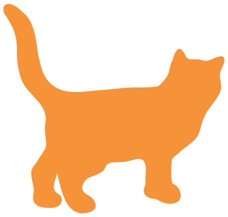 the executive | 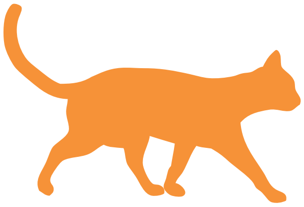 sidekick | 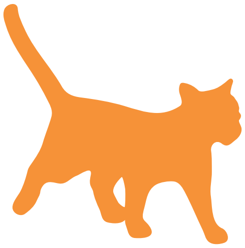 personal assistant |
| high valiance (29–43) | 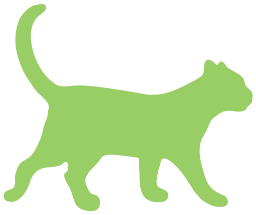 MVP | 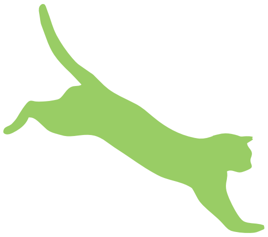 party animal | 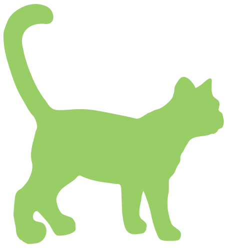 leader of the band |
