# Supplementary material for: Feline-Ality™ in Real Life: A Program Evaluation
Source: Animals (Basel). 2023 Aug 29;13(17):2752. doi: 10.3390/ani13172752 (PMC10486580; doi:10.3390/ani13172752)
Supplement: Supplementary file 1 [file animals-13-02752-s001.zip › Figure S3.pdf]

cat adopter survey

MYM
MEET YOUR MATCH®

first name	last name	date
address		apt. #
city	state	zip
home phone ( )	work phone ( )	email

1	I would consider my household to be like	A library	Middle of the road	A carnival	
2	I am comfortable with a cat that likes to play "chase my ankles" and similar games	No	Somewhat	Yes	
3	I want my cat to interact with guests that come to my house	Little of the time	Some of the time	All of the time	
4	How do you feel about a boisterous cat that gets into everything?	Love them but rather not to live with them	Depends on the situation	Fine by me	
5	My cat needs to be able to adjust to new situations quickly	Not important	Somewhat	Yes	
6	I want my cat to love being with children in my home	It's not important whether my cat loves being with children	Some of the time	Most of the time	Children do not often come to my house
7	My cat needs to be able to be alone	More than 9 hours per day	4 to 8 hours per day	Less than 4 hours per day	
8	When I am at home, I want my cat to be by my side or in my lap	Little of the time	Some of the time	All of the time	
9	I want my cat to enjoy being held	Little of the time	Some of the time	Most of the time	
10	I need my cat to get along with (circle all that apply)				Dogs Cats Birds Other
11	My cat will be	Inside	Inside and Outside	Outside	
12	I have lived with cats before	No		Yes Date ________	Currently
13	I prefer my cat to be talkative	No		Yes	It's not important if my cat is talkative
14	I want my cat to play with toys	Little of the time	Sometimes	Often	
15	I want my cat to be active	Not very active at all	Somewhat	Yes, very	
16	It is most important to me that my cat ______________________ (fill in the blank)				

**FOR OFFICE USE ONLY**	**RECOMMENDED COLOR MATCH: PURPLE ORANGE GREEN**<br>**RECOMMENDED FELINE-ALITY™(IES)** ______________________

Copyright © 2009 The American Society for the Prevention of Cruelty to Animals®. Reprinted with permission of the ASPCA®.
