# Supplementary material for: Feline-Ality™ in Real Life: A Program Evaluation
Source: Animals (Basel). 2023 Aug 29;13(17):2752. doi: 10.3390/ani13172752 (PMC10486580; doi:10.3390/ani13172752)
Supplement: Supplementary file 1 [file animals-13-02752-s001.zip › Figure S4.pdf]

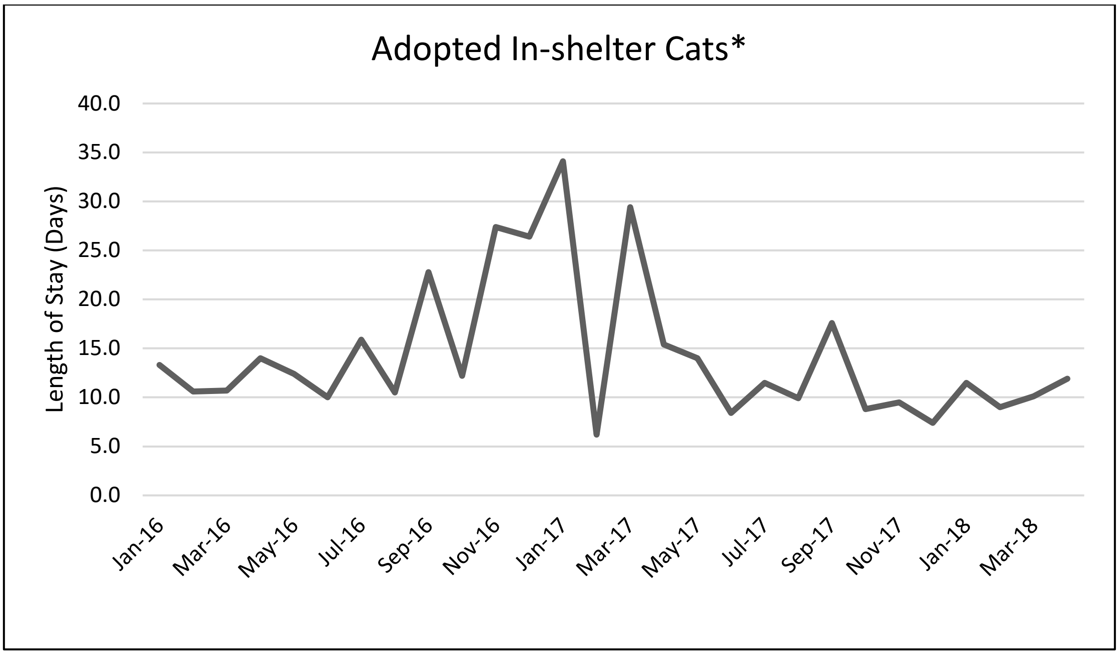

Monthly averages in LOS for all cats over the age of 6 months were visually compared from January 2016 through March 2018 (see Figure S4). Based on the variance gleaned from the data presented in this chart, we were able to investigate variability by comparing the variance in LOS across the months prior to the introduction Feline-ality™ and subsequent to its introduction. This was done in three ways: removing the most extreme values that might be a statistical side effect of regression to the mean (January 2017 being the most extreme LOS duration (and therefore perhaps evidence of the impetus for instituting a program at all) and February of 2017, the extreme nadir); comparing the same months to each other between the two years (including extremes), or splitting the data at February of 2017, the month in which the program was formally introduced (including extremes).

In our post hoc analysis, we looked at these data in all three ways listed above and applied Levene's test of homogeneity of variance to each; all were non-significant (p values: .61, .56, and .31 respectively), which indicates that variability did not differ between the time prior to Feline-ality™ and the time after (the Levene test proposes the null hypothesis that variance between two groups is the same. If the p value for the test is significant, then that null hypothesis is violated and there is evidence that the variances of the two groups are in fact different).
