# Supplementary material for: Feline-Ality™ in Real Life: A Program Evaluation
Source: Animals (Basel). 2023 Aug 29;13(17):2752. doi: 10.3390/ani13172752 (PMC10486580; doi:10.3390/ani13172752)
Supplement: Supplementary file 1 [file animals-13-02752-s001.zip › Figure S5.pdf]

Ideally, in order to make inferences about the effect of Feline-ality™, a true experimental design would be employed. However, as is common in real-world applications, the level of experimental control required to maintain the sufficiently high internal validity necessary to make causal inferences and extrapolate results to a broader population was not an option. With the institution of corporate or governmental programs, the researcher most often does not control who is exposed to the intervention, removing one keystone of experimental designs: random assignment to treatment groups. Quasi-experimental designs are those that resemble experimental designs in structure and include additional elements to attempt to mitigate the potential effects due to the lack of randomization. For an excellent explanation and classic examples of this type of design, see Campbell and Ross [29].

Non-experimental designs are subject to threats to internal validity; that is to say, identifying whether the intervention in question actually caused any observed effect. Quasi-experimental designs are able to address some of these threats to internal validity to varying degrees. The main threats to internal validity, or inferring causality, are: maturation, testing, instrumentation, regression to the mean, selection, history, and attrition [15-17]. Testing threats do not apply to our study (there were not multiple tests administered to participants that could have had an effect on subsequent tests). The time-series itself (more than one pre- and post-test period) allowed us to look for any possible threats due to regression to the mean. Had there been evidence of regression to the mean, we would expect to see a peak high in euthanasia, and a nadir in adoptions prior to the implementation of the new program (these metrics would have been the triggers for such an implementation); we did not see this pattern and it is therefore safe to say that regression to the mean does not explain our findings.

An instrumentation threat was also a possibility in our study: the computer system used for tracking animals changed after 2015. According to the shelter, the length of stay information collected in 2015 using that database was potentially faulty [23]. However, the multiple time points over the subsequent years and the lack of significant changes in length of stay across subsequent years, support the idea that instrumentation issues were not a significant problem in our study. It is possible that the change in computer database might also have affected other outcome variables; post hoc analysis of all variables, omitting all data from 2015, produced the same patterns of significance and lack thereof for all outcome variables.

Our study was also able to address possible instrumentation effects and investigate possible maturation or history effects (internal or external processes or events that might have occurred during the treatment period or cooccurred with the treatment), as well as selection threats (our study shelter being markedly different from the outset from other shelters), by including a non-equivalent control group. Our non-equivalent control group was the Multnomah County Animal Shelter (MCAS). Though similar to our primary shelter (MCAS is a municipal open-admission shelter located in Portland, Oregon), there were some important non-equivalencies that should be mentioned.

We limited our primary dataset to only cats that might have been theoretically affected by Feline-ality™ (e.g., over the age of 9 months, not feral, etc.—see previous subjects description for details); we were unable to access that detailed information for the MCAS dataset. Also, our primary dataset counted outcomes, not individual cats (some cats who visited the shelter more than once had different outcomes at each visit, as described previously). Therefore, there were more outcomes than unique cats (on average, 5% more outcomes than cats across the five years), which appears to differ from the comparison group. With these caveats, and bearing in mind that a non-equivalent control group is exactly that, nevertheless, the additional data from MCAS were considered useful to look at broad trends in outcomes across years in a similar shelter. Instrumentation effects are unlikely—the trends in outcome variables that we were able to compare are similar and would be expected to be different if the change in computer database at our primary shelter (SAS) were a large problem. Selection threats cannot be definitively ruled out, as the starting percentages of adoptions and transfers were markedly different (e.g., about half of cats were adopted at MCAS and approximately 40% were transferred out of MCAS, whereas at our primary shelter, those same outcomes were approximately 85% and 10%, respectively).
