# Supplementary material for: Feline-Ality™ in Real Life: A Program Evaluation
Source: Animals (Basel). 2023 Aug 29;13(17):2752. doi: 10.3390/ani13172752 (PMC10486580; doi:10.3390/ani13172752)
Supplement: Supplementary file 1 [file animals-13-02752-s001.zip › Table S1.pdf]

Item	Observer instructions		Listed possible behaviors	Scores
1. Body Posture	Quietly approach the cat's cage door and while standing in front of the cat's cage, take a quick mental snapshot of the cat's body posture. Choose one of the following Reponses.		Soft and relaxed	1
			Tense body with twitching tail	1
			Flattened body with dilated pupils	-1
2. Greeting Approach	Immediately after taking a mental snapshot of the cat's body posture during item #1, begin to speak to the cat in a soft, normal tone of voice. Observe the cat's response to your presence through the closed cage door. Choose one of the following responses.		At front of cage, soliciting attention by rubbing, chirping, etc.	3
			Comes to the front of the cage after you encourage	2
			Does not approach but meows, chirps, or blinks (circle all that apply)	1
			Does not approach	0
			Attempts to hide	-1
			Hisses or growls	-2
			Charges	-3
3. Cage Condition	While quietly standing in front of the cat's cage, observe and note its condition. Choose one of the following.		Bedding/cage paper moved, cat hiding under	-1
			Cage rearranged, cat on top or not hiding	1
			No change	0
			Other, please describe	0
4. Social Response when door is opened	After completing items 1 through 3, calmly and slowly open the cage door while watching the cat. This should be done without talking. Observe the cat at the moment the door is initially opened. Choose one of the following responses.		Remains relaxed and soft, approaches me	1
			Remains relaxed and soft, does not approach	0
			Becomes stiff with tight tail flicks and standing	1
			Crouches, body stiff	-1
5a-b. Introduction to a Novel Room	Take the cat out of the cage and place him into a plastic crate (cleaned and allowed to air) for transport to the Novel Room. Ideally the cat should be loaded into the crate from above (turn crate so door is facing the ceiling). Throughout this item, you will be recording the amount of time the cat is either interacting or not interacting with you. In order to obtain an accurate assessment of time spent in each activity, use two stopwatches-one to keep the total time (start when you open crate door, end at five minutes), and the second to keep track of time interacting with you. Click on the second stopwatch whenever an interaction begins and click it off once the interaction ends. IMPORTANT: OTHER THAN EYE CONTACT, DO NOT INITIATE, ENGAGE WITH OR RESPOND TO THE CAT. INTERACTION INCLUDES EYE CONTACT OR OTHER BEHAVIOR EVEN WHEN THE CAT IS IN THE CARRIER. Interactive behaviors include meows, chirps, looks, blinks, rubbing against you, touching you, and being in your lap. Be sure to include those behaviors made while inside the carrier after the door has been opened.	5a	(5a) Exits carrier in 25 seconds or less with a tall body posture	1
			(5a) Exits the carrier in 25 seconds or less with crouched body posture	0.5
			(5a) Exits the carrier within 25 seconds and quickly scoots to hiding place, keeping body low to the ground	-0.5
		5a - choose one (if applicable)	Investigates room while standing tall	1
			Investigates room in a low body posture	0.5
			Races out of carrier to one spot in the room and stays there	-0.5
		5b	(5b) Time spent interacting with evaluator (as determined by second stopwatch)	__sec
			(5b) Time not spent interacting with the evaluator	__sec
		5b - choose one	Interacts for more than 60 seconds	1 1/2
			Interacts for 30-60 seconds	1
			Interacts for less than 30 seconds	0
6. Call and Approach	Crouch down on the opposite side of the room from the cat and about five to six feet away, start the stopwatch, and call the cat several times. Extend one hand, closed in a fist, toward him. Stop the stopwatch when the cat approaches and makes contact with your hand or body. Allow the cat up to 30 seconds to approach. Be sure to talk softly and encourage forward with voice. Check all behaviors that occur.		Makes eye contact	1
			Does not make eye contact	-!
			Approaches	3
			Sniffs or head butts	3
			Rolls on back or rolls over	2
			Meows, purrs and/or chirps (circle all that apply)	1
			Watches with no approach	0
			Retreats	-1
			Hisses and/or growl	-2
			Sniffs or head butts	3
			licks or rubs on hand	3

7. Open Hand	While still crouching, extend an open hand to the cat. Your hand should be lower than the cat's head. If the cat did not approach you in item #6, walk toward and crouch next to the cat. Check all behaviors that occur.	Rolls on back or rolls over	2
		Meows, purrs and/or chirps (circle all that apply)	2
		Retreats/defensive position	-2
		Hisses and/or growls	-2
		Swats/attempts to swat hand	-2
		Bites/attempts to bite hand	-3
8. Stroking	While talking to the cat, use your open hand, slightly cupped, to stroke the cat in long strokes along the head, back and sides. Continue for 4 to 5 long slow strokes if possible. Check all behaviors that occur.	Rubs against legs or hand	3
		Head butts	3
		Circles you attentively	2
		Meows, purrs and/or chirps (circle all that apply)	2
		Rolls on back or rolls over	2
		Shows initial fear but then relaxes	0
		Retreats/defensive position	-1
		Hisses and/or growls	-2
		Swats or attempts to swat hand	-2
		Bites or attempts to bite hand	-3
9. Play	Beginning a couple of feet away from the cat, slowly move a piece of string/yarn along the floor to initiate play. Observe that cat's reaction. Then try to engage the cat in play with two other toys from the following list (for a total of three different toys): Ball, Toy Mouse, Cat Charmer, and Feather Wand. Do not use catnip toys during the Feline-ality Assessment. Check all behaviors that occur. Circle the toy preferences on the score sheet so the	Watches toy intently	3
		Chases toy	3
		Comes back for stroking	2
		Ignores toys	0
		Attends to something else	-1
		Avoids eye contact	-1
10. Hug	Call the cat again until he approaches, or approach him slowly yourself. Begin to stroke the cat again and if he is calm, gently pick him up and cradle him against your chest, his body sideways to your chest for a full two seconds. Check all behaviors that occur.	Is relaxed	3
		Extends paw to evaluator's neck or shoulder in an affiliative manner	3
		Meows, purrs and/or chirps (circle all that apply)	2
		Accepts hold but remains a bit tense	2
		Struggles to escape	-1
		Hisses/growls	-2
		Stiffens and extends claws	-2
		Swats/attempts to swat hand	-3
		Bites/attempts to bite hand	-3
11. Sensitivity	Sit on the ground an allow the cat to approach if he chooses. Wait at least 60 seconds before you stroke the cat along his back and gently grasp the cat's tail firmly at the base and pull up with a steady pressure just enough almost, but not quite, move his back feet off the floor. Hold for 1 second. Check all behaviors that occur.	Rolls on back or rolls over	3
		Shows no reaction	3
		Meows, purrs and/or chirps (circle all that apply)	1
		Struggles/tries to escape	0
		Hisses/growls	-1
		Swats/attempts to swat	-2
		Bites/attempts to bite	-3

overall score		
item #1		
item #2		
item #3		
item #4		
item #5a		
item #5b		
item #6		
item #7		
item #8		
item #9		
item #10		
item #11		
	independent-gregarious	valiance
total score		
